# Influence of Newtonian Heating on Three Dimensional MHD Flow of Couple Stress Nanofluid with Viscous Dissipation and Joule Heating

**DOI:** 10.1371/journal.pone.0124699

**Published:** 2015-04-14

**Authors:** Muhammad Ramzan

**Affiliations:** Department of Mathematics, College of Science, Al-Zulfi, Majmaah University, Saudi Arabia; North China Electric Power University, CHINA

## Abstract

The present exploration discusses the influence of Newtonian heating on the magnetohydrodynamic (MHD) three dimensional couple stress nanofluid past a stretching surface. Viscous dissipation and Joule heating effects are also considered. Moreover, the nanofluid model includes the combined effects of thermophoresis and Brownian motion. Using an appropriate transformation, the governing non linear partial differential equations are converted into nonlinear ordinary differential equations. Series solutions using Homotopy Analysis method (HAM) are computed. Plots are presented to portrait the arising parameters in the problem. It is seen that an increase in conjugate heating parameter results in considerable increase in the temperature profile of the stretching wall. Skin friction coefficient, local Nusselt and local Sherwood numbers tabulated and analyzed. Higher values of conjugate parameter, Thermophoresis parameter and Brownian motion parameter result in enhancement of temperature distribution.

## 1 Introduction

The conventional heat transfer fluids such as oil, water, ethylene glycol etc. with solid nanoparticles of size 1–100 nm are known as nanofluids. Such nanoparticles are structured as different types of nanomaterials like *Au*, *Ag*, *Cu* metals, *CuO*, *TiO*_2_and *Al*_2_ *O*_3_. Nanofluids are utilized to attain the maximum enhancement in the thermal characteristics under minimum concentrations. The thermal conductivity of the base fluid is twice by submerging the nanoparticles into that base fluid [1]. Nanofluids are usually involved in cancer therapy, safer surgery, coolants of nuclear reactors, transformer cooling and vehicle computers. Nowadays, it is found that nanofluid is considered as a best candidate in nuclear reactor safety problems. Azizian et al. [2] explored that the nanofluids are used in designing the waste heat removal equipment. Further, the magneto nanofluid has a great advancement in the manufacturing processes because of its diverse applications in biomedical such as wound treatment, sterilized devices, gastric medications and many others. It is well known fact that the magnetic field is utilized for manipulation of electrically conducting nanofluids due to which the desired effects in applications can be obtained. The magneto nanofluids have great importance in the processes of targeted drug release, elimination of tumors with hyperthermia, asthma treatment, synergistic effects in immunology etc. Some recent investigations on nanofluids and magneto nanofluids can be seen in the references [3–6] and many therein.

Flows of non-Newtonian fluids with heat transfer are fairly significant in numerous industrial processes like multiphase mixtures, natural products, biological fluids, food products and agricultural and dairy wastes. Particularly the attention of recent researchers in such flows induced by stretching surface has increased due to their usage and broad range of applications. Attention has been mainly focused to the control of quality of final product in various manufacturing and processing industries such as hot rolling, wire drawing, continuous casting, glass fiber and paper production. A variety of chemical engineering processes regarding polymer extrusion and in metallurgy engage cooling of a molten liquid being stretched into a cooling systems. The final product of material depend upon two aspects. One is the cooling liquid used and the other is rate of stretching. Stretching rate is important because sudden solidification occurs due to rapid stretching property. Crane [7] was the first who constructed the closed form solution for viscous stretched flow. Afterwards, flow analysis by stretching surface has been explored through diverse aspects (see recent attempts [8–11]).

The characteristics of MHD in flow analysis are vital from many engineering and industrial applications point of view. The application of such flow characteristics are common in design cooling systems with liquid metals, nuclear reactors, MHD generators, accelerators, blood flow measurements, pumps and flow meters. In view of such applications many investigators considered the characteristics of MHD in flows generated by stretching surface. MHD flow of an incompressible fluid over a moving surface is studied by Makinde [12]. Zheng et al. [13] presented the flow of MHD and heat transfer over a porous surface with velocity slip and temperature jump conditions. Hayat et al. [14] considered the three-dimensional MHD flow with heat and mass transfer in a porous medium. MHD flow of UCM fluid over a porous stretching sheet is analyzed by Raftari and Yildrim [15]. Seddeek et al. [16] examined the MHD flow by a wedge. Ishak et al. [17] examined the MHD flow stagnation point on a vertical permeable surface. Unsteady MHD flow of an impulsively rotating and translating sphere in presence of buoyancy forces is presented by Dinarvand et al. [18].

Much attention in the past has been given to study the stretched flows with heat transfer either through constant wall temperature or constant wall heat flux. Besides this, there is another class of flow problems in which the rate of heat transfer is proportional to the local surface temperature from the bounding surface with finite heat capacity which is known as Newtonian heating or conjugate convective flow. Merkin [19] presented the boundary layer natural convection flow by a vertical surface with Newtonian heating. Exact solution for unsteady free convection flow past an impulsive vertical surface in the presence of Newtonian heating is obtained by Chaudhary et al. [20].

The aim here is to discuss the effect of Newtonian heating in the three dimensional flow of couple stress nanofluid with viscous dissipation and Joule heating. To our knowledge this seems a first attempt in this direction. The problem formulation is given using fundamental laws of mass, linear momentum and energy. Convergent series solutions by the homotopic approach are constructed [21–25]. The velocity components, temperature, and concentration, are illustrated graphically. However, skin friction, Nusselt number and sherwood numbers are tabulated numerically against different parameters is also examined.

## 2 Formulation

**Fig 1:**
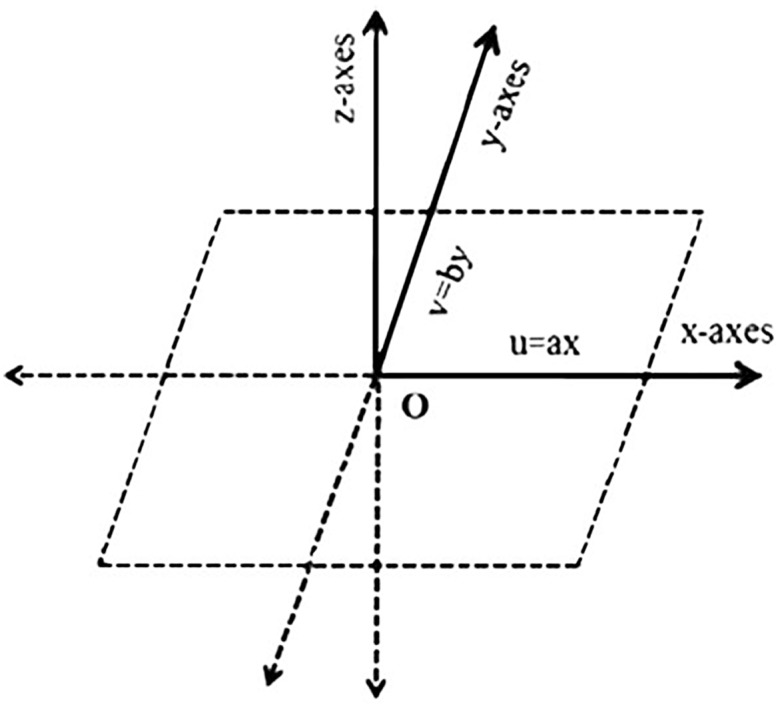
Physical Flow.

We investigate the three dimensional incompressible flow of couple stress nanofluid past a stretching surface with Newtonian heating. Here we assumed that *u* = *ax* and *v* = *by* are the stretching velocities of the sheet in the *x* and *y*-axes respectively which vary linearly from the leading edge. The fluid is electrically conducting in the presence of a uniform applied magnetic field. Effects of viscous dissipation and Joule heating are taken into account. Induced magnetic field is neglected subject to assumption of small magnetic Reynolds number. The electric field is not taken into account. Heat transfer analysis is examined in the presence of Newtonian heating. The geometrical configuration of the present flow is shown in Fig 1. The governing boundary layer three dimensional equations are [11]:

$$\begin{array}{c} \frac{\partial u}{\partial x}+\frac{\partial v}{\partial y}+\frac{\partial w}{\partial z}=0, \end{array}$$ (1)

$$\begin{array}{c} u\frac{\partial u}{\partial x}+v\frac{\partial u}{\partial y}+w\frac{\partial u}{\partial z}=\nu \frac{\partial^{2}u}{\partial z^{2}}-\nu^{\prime }\frac{\partial^{4}u}{\partial z^{4}}-\frac{\sigma B_{0}^{2}}{\rho }u, \end{array}$$ (2)

$$\begin{array}{c} u\frac{\partial v}{\partial x}+v\frac{\partial v}{\partial y}+w\frac{\partial v}{\partial z}=\nu \frac{\partial^{2}v}{\partial z^{2}}-\nu^{\prime }\frac{\partial^{4}v}{\partial z^{4}}-\frac{\sigma B_{0}^{2}}{\rho }v, \end{array}$$ (3)

$$\begin{array}{ccc} u\frac{\partial T}{\partial x}+v\frac{\partial T}{\partial y}+w\frac{\partial T}{\partial z} & = & \frac{k}{\rho c_{p}}(\frac{\partial^{2}T}{\partial z^{2}})+\frac{2\mu }{\rho c_{p}}[{\left(\frac{\partial u}{\partial z}\right)}^{2}+{\left(\frac{\partial v}{\partial z}\right)}^{2}]+\\ & & \frac{n}{\rho c_{p}}[{\left(\frac{\partial^{2}u}{\partial z^{2}}\right)}^{2}+{\left(\frac{\partial^{2}v}{\partial z^{2}}\right)}^{2}]+\frac{\sigma B_{0}^{2}}{\rho c_{p}}\left(u^{2}+v^{2}\right)\\ & & +\;\tau [D_{B}\frac{\partial C}{\partial z}\frac{\partial T}{\partial z}+\frac{D_{T}}{T_{\infty }}{\left(\frac{\partial T}{\partial z}\right)}^{2}], \end{array}$$ (4)

$$\begin{array}{c} u\frac{\partial C}{\partial x}+v\frac{\partial C}{\partial y}+w\frac{\partial C}{\partial z}=D_{B}(\frac{\partial^{2}C}{\partial z^{2}})+\frac{D_{T}}{T_{\infty }}(\frac{\partial^{2}T}{\partial z^{2}}). \end{array}$$ (5)

with the following boundary conditions:

$$\begin{array}{c} u=ax,\;\;\;v=by,\;\;\;w=0,\;\;\;\frac{\partial T}{\partial z}=-h_{s}T,\;C=C_{w}\;\text{at}\;\;\;\;z=0, \end{array}$$

$$\begin{array}{c} u\to 0,\;\;v\to 0,\;\;\;\frac{\partial u}{\partial z}\to 0,\;\;\;\frac{\partial v}{\partial z}\to 0,\;\;\;T\to T_{\infty },\;C\;\to C_{\infty }\;\;\text{as}\;\;\;\;z\to \infty . \end{array}$$ (6)

Newtonian heating in which heat transfer from bounding surface with a finite heat capacity is proportional to the local surface temperature. Recently Newtonian heating effects have been utilized by different researchers due to their practical applications such as to design heat exchanger, conjugate heat transfer around fins and also in convection flows setup where bounding surfaces absorb heat by solar radiations.

In the above equations *u*, *v* and *w* are the velocity components in the *x*, *y* and *z* directions, $\nu =\frac{\mu }{\rho }$ the kinematic viscosity, $\nu^{\prime }=\frac{n}{\rho },$ the couple stress viscosity, *a* and *b* are dimensional rate constants, *ρ* the density, *σ* the electric charge density, *B*_0_ the applied magnetic field, *h*_*s*_ the heat transfer coefficient, *n* the couple stress vicosity parameter, *T* and *T*_∞_ the fluid and ambient temperatures respectively, *k* the thermal conductivity, *τ* ratio between the effective heat capacity of the nano particle material and heat capacity of the fluid and *c*_*p*_ the specific heat, *D*_*B*_ the Brownian diffusion coefficient, *D*_*T*_ the thermophoretic diffusion coefficient, *C* the concentration field. On setting

$$\begin{array}{ccc} \eta & = & \sqrt{\frac{a}{v}}z,\;u=axf^{\prime }\left(\eta \right),\;v=ayg^{\prime }\left(\eta \right),\;w=-\sqrt{a\nu }\left(f\left(\eta \right)+g\left(\eta \right)\right),\;\\ \theta (\eta ) & = & \frac{T-T_{\infty }}{T_{\infty }},\;\phi (\eta )=\frac{C-C_{\infty }}{C_{w}-C_{\infty }}, \end{array}$$ (7)

incompressibility condition (Eq 1) is satisfied and Eqs (2) to (6) can be put into the forms

$$\begin{array}{c} f^{\prime \prime \prime }+\left(f+g\right)f^{\prime \prime }-f^{\prime 2}-Kf^{\prime \prime \prime \prime \prime }-M^{2}f^{\prime }=0, \end{array}$$ (8)

$$\begin{array}{c} g^{\prime \prime \prime }+\left(f+g\right)g^{\prime \prime }-g^{\prime 2}-Kg^{\prime \prime \prime \prime \prime }-M^{2}g^{\prime }=0, \end{array}$$ (9)

$$\begin{array}{ccc} & & \theta^{\prime \prime }+Prf\theta^{\prime }+Prg\theta^{\prime }+2\mathrm{Pr}Ec\left(f^{\prime \prime 2}+L^{2}g^{\prime \prime 2}\right)-K\mathrm{Pr}Ec\left(f^{\prime \prime \prime 2}+L^{2}g^{\prime \prime \prime 2}\right)+\mathrm{Pr}M^{2}Ec\left(f^{\prime 2}+L^{2}g^{\prime 2}\right)\\ & & \;+\mathrm{Pr}Nb\theta^{\prime }\phi^{\prime }+\mathrm{Pr}Nt\theta^{\prime 2}=0, \end{array}$$ (10)

$$\begin{array}{c} \phi^{\prime \prime }+(\frac{N_{t}}{N_{b}})\theta^{\prime \prime }+\mathrm{Pr}Le(f+g)\phi^{\prime }=0, \end{array}$$ (11)

$$\begin{array}{ccc} f & = & 0,\;g=0,\;f^{\prime }=1,\;g^{\prime }=\beta \text{,}\;\theta^{\prime }=-\gamma \left(1+\theta \left(0\right)\right),\phi \left(0\right)=1\;\;\;\text{at}\;\eta =0,\\ f^{\prime }\left(\infty \right) & \to & 0,\;g^{\prime }\left(\infty \right)\to 0,\;\theta \to 0,\;f^{\prime \prime }\left(\infty \right)\to 0,\;g^{\prime \prime }\left(\infty \right)\to 0,\;\theta \left(\infty \right)\to 0,\phi \left(\infty \right)\to 0, \end{array}$$ (12)

in which the prime signifies differentiation with respect to *η*. The *K* is the dimensionless couple stress parameter, *M* the Hartman number, *β* the ratio of rates parameter, Pr the Prandtl number, *Ec* is the Eckert number, *L* is the dimensionless parameter, *N*_*b*_ Brownian motion parameter, *N*_*t*_ thermophoresis, *Le* Lewis number and *γ* the conjugate parameter for Newtonian heating. These parameters are defined as follows:

$$\begin{array}{ccc} K & = & \frac{v^{\prime }a}{v^{2}},\;\;\;\;Pr=\frac{\mu c_{p}}{k},\;\;\;\;M^{2}=\frac{\sigma B_{o}^{2}}{\rho a}\text{,}\;\;\;\gamma =h_{s}\sqrt{\frac{\nu }{a}}\text{,}\;\;\;\;\beta =\frac{b}{a}\text{,}\;\;Le=\frac{\alpha }{D_{B}}\\ Ec & = & \frac{u_{w}^{2}}{c_{p}T_{\infty }},\;\;\;\;L=\frac{y}{x}\text{,}\;\;\;\;\;\;N_{b}=\frac{\tau D_{B}}{\nu }\left(C_{w}-C_{\infty }\right),\;\;Nt=\frac{\tau D_{T}}{\nu }. \end{array}$$

Skin friction, local Nusselt number and Sherwood number are given by

$$\begin{array}{c} C_{f}Re_{x}^{1/2}=f^{\prime \prime }(0)-Kf^{\prime \prime \prime \prime }\left(0\right),\;\;\;\;\;C_{g}Re_{x}^{1/2}=g^{\prime \prime }(0)-Kg^{\prime \prime \prime \prime }\left(0\right), \end{array}$$ (13)

$$\begin{array}{c} Nu/Re_{x}^{1/2}=\gamma (1+\frac{1}{\theta (0)}), \end{array}$$ (14)

$$\begin{array}{c} \;Sh{Re}_{x}^{-1/2}=-\phi^{\prime }(0), \end{array}$$ (15)

where *Re*_*x*_ = *ux*/*ν* is the local Reynolds number.

## 3 Series solutions

Homotopy analysis method was first proposed by Liao [21] in 1992 which is used for the construction of series solution of highly nonlinear problems. It is preferred over the other methods due to the following advantages.

- It does not depend upon the small or large parameters.
- It ensures the convergence of series solutions.
- It provides us great choice to select the base function and linear operator.

The series solutions by homotopy analysis method requires the initial guesses and linear operators in the following forms:

$$\begin{array}{c} f_{0}\left(\eta \right)=(1-e^{-\eta }),\;g_{0}\left(\eta \right)=\beta (1-e^{-\eta }),\;\theta_{0}\left(\eta \right)=\frac{\gamma \exp(-\eta )}{1-\gamma },\;\phi_{0}(\eta )=\exp(-\eta ), \end{array}$$ (16)

$$\begin{array}{c} {𝓛}_{f}=f^{\prime \prime \prime }-f^{\prime },\;{𝓛}_{g}=g^{\prime \prime \prime }-g^{\prime },\;\;{𝓛}_{\theta }=\theta^{\prime \prime }-\theta .{𝓛}_{\phi }(\eta )=\phi^{\prime \prime }-\phi . \end{array}$$ (17)

The auxiliary linear operators have the following properties

$$\begin{array}{c} {𝓛}_{f}\left(A_{1}+A_{2}e^{\eta }+A_{3}e^{-\eta }\right)=0,\;{𝓛}_{g}\left(A_{4}+A_{5}e^{\eta }+A_{6}e^{-\eta }\right)=0,\;{𝓛}_{\theta }\left(A_{7}e^{\eta }+A_{8}e^{-\eta }\right)=0,{𝓛}_{\phi }\left(A_{9}e^{\eta }+A_{10}e^{-\eta }\right)=0, \end{array}$$ (18)

in which *A*_*i*_ (*i* = 1 − 10) are the arbitrary constants.

The zeroth order deformation problems can be expressed as follows:

$$\begin{array}{c} (1-p){𝓛}_{f}[\hat{f}\left(\eta ;p\right)-f_{0}\left(\eta \right)]=p\hbar_{f}{𝓝}_{f}[\hat{f}\left(\eta ;p\right),\hat{g}\left(\eta ;p\right)], \end{array}$$ (19)

$$\begin{array}{c} (1-p){𝓛}_{g}[\hat{g}\left(\eta ;p\right)-g_{0}\left(\eta \right)]=p\hbar_{g}{𝓝}_{g}[\hat{f}\left(\eta ;p\right),\hat{g}\left(\eta ;p\right)], \end{array}$$ (20)

$$\begin{array}{c} (1-p){𝓛}_{\theta }[\hat{\theta }\left(\eta ;p\right)-\theta_{0}\left(\eta \right)]=p\hbar_{\theta }{𝓝}_{\theta }[\hat{f}\left(\eta ;p\right),\hat{g}\left(\eta ;p\right),\hat{\theta }\left(\eta ,p\right)], \end{array}$$ (21)

$$\begin{array}{c} (1-p){𝓛}_{\phi }[\hat{\phi }(\eta ;p)-\phi_{0}(\eta )]=p\hbar_{\phi }{𝓝}_{\phi }[\hat{\phi }(\eta ;p),\hat{f}(\eta ;p),\hat{\theta }(\eta ;p)], \end{array}$$ (22)

$$\begin{array}{c} \hat{f}\left(0;p\right)=0,\;{\hat{f}}^{\prime }\left(0;p\right)=1,\;{\hat{f}}^{\prime }\left(\infty ;p\right)=0,\;\hat{g}\left(0;p\right)=0,\;{\hat{g}}^{\prime }\left(0;p\right)=\beta ,\;{\hat{g}}^{\prime }\left(\infty ;p\right)=0, \end{array}$$ (23)

$$\begin{array}{c} {\hat{f}}^{\prime \prime }\left(\infty ;p\right)=0,\;{\hat{g}}^{\prime \prime }\left(\infty ;p\right)=0,\;{\hat{\theta }}^{\prime }\left(0,p\right)=-\gamma \left[1+\theta \left(0,p\right)\right],\;\hat{\theta }\left(\infty ,p\right)=0,\;\;\hat{\phi }(0;p)=1,\;\;\;\;\;\hat{\phi }\;(\infty ;p)=0, \end{array}$$ (24)

$$\begin{array}{ccc} {𝓝}_{f}\left[\hat{f}\left(\eta ,p\right),\hat{g}\left(\eta ,p\right)\right] & = & \frac{\partial^{3}\hat{f}\left(\eta ,p\right)}{\partial \eta^{3}}-{\left(\frac{\partial \hat{f}\left(\eta ,p\right)}{\partial \eta }\right)}^{2}+\\ & & \left(\hat{f}\left(\eta ,p\right)+\hat{g}\left(\eta ,p\right)\right)\frac{\partial^{2}\hat{f}\left(\eta ,p\right)}{\partial \eta^{2}}-K\;{\hat{f}}^{\prime \prime \prime \prime \prime }\left(\eta ,p\right)-M^{2}\frac{\partial \hat{f}\left(\eta ,p\right)}{\partial \eta }, \end{array}$$ (25)

$$\begin{array}{ccc} {𝓝}_{g}\left[\hat{f}\left(\eta ,p\right),\hat{g}\left(\eta ,p\right)\right] & = & \frac{\partial^{3}\hat{g}\left(\eta ,p\right)}{\partial \eta^{3}}-{\left(\frac{\partial \hat{g}\left(\eta ,p\right)}{\partial \eta }\right)}^{2}+\\ & & \left(\hat{f}\left(\eta ,p\right)+\hat{g}\left(\eta ,p\right)\right)\frac{\partial^{2}\hat{g}\left(\eta ,p\right)}{\partial \eta^{2}}-K\;{\hat{g}}^{\prime \prime \prime \prime \prime }\left(\eta ,p\right)-M^{2}\frac{\partial \hat{g}\left(\eta ,p\right)}{\partial \eta }, \end{array}$$ (26)

$$\begin{array}{ccc} {𝓝}_{\theta }\left[\hat{\theta }\left(\eta ,p\right),\hat{f}\left(\eta ,p\right),\hat{g}\left(\eta ,p\right)\right] & = & \frac{\partial^{2}\hat{\theta }\left(\eta ,p\right)}{\partial \eta^{2}}+\mathrm{Pr}(\begin{array}{c} \hat{f}\left(\eta ,p\right)+\\ \hat{g}\left(\eta ,p\right) \end{array})\frac{\partial \hat{\theta }\left(\eta ,p\right)}{\partial \eta }+2\mathrm{Pr}Ec(\begin{array}{c} {\left(\frac{\partial^{2}\hat{f}\left(\eta ,p\right)}{\partial \eta^{2}}\right)}^{2}+\\ L^{2}{\left(\frac{\partial^{2}\hat{g}\left(\eta ,p\right)}{\partial \eta^{2}}\right)}^{2} \end{array})\\ & & -K\mathrm{Pr}Ec(\begin{array}{c} \frac{\partial^{3}\hat{f}\left(\eta ,p\right)}{\partial \eta^{3}}+\\ L^{2}\frac{\partial^{3}\hat{g}\left(\eta ,p\right)}{\partial \eta^{3}} \end{array})+\mathrm{Pr}M^{2}Ec(\begin{array}{c} {\left(\frac{\partial \hat{f}\left(\eta ,p\right)}{\partial \eta }\right)}^{2}+\\ L^{2}{\left(\frac{\partial \hat{g}\left(\eta ,p\right)}{\partial \eta }\right)}^{2} \end{array})\\ & & +\mathrm{Pr}(N_{b}\frac{\partial \hat{\theta }(\eta ;p)}{\partial \eta }\frac{\partial \hat{\phi }\left(\eta ,p\right)}{\partial \eta }+N_{t}\frac{\partial^{2}\hat{\theta }\left(\eta ,p\right)}{\partial \eta^{2}}), \end{array}$$ (27)

$$\begin{array}{ccc} {𝓝}_{\phi }\hat{f}(\eta ;p),\hat{g}\left(\eta ,p\right),\hat{\theta }(\eta ;p),\hat{\phi }(\eta ;p) & = & \frac{\partial^{2}\hat{\phi }\left(\eta ,p\right)}{\partial \eta^{2}}+\mathrm{Pr}Le[\hat{f}(\eta ;p)+\hat{g}\left(\eta ,p\right)]\frac{\partial \hat{\phi }\left(\eta ,p\right)}{\partial \eta }\\ & & +\frac{N_{t}}{N_{b}}\frac{\partial^{2}\hat{\phi }\left(\eta ,p\right)}{\partial \eta^{2}}, \end{array}$$ (28)

where *p* is an embedding parameter, ℏ_*f*_, ℏ_*g*_, ℏ_*θ*_ and ℏ_*ϕ*_ are the non-zero auxiliary parameters and 𝓝_*f*_, 𝓝_*g*_, 𝓝_*θ*_and 𝓝_*ϕ*_ the nonlinear operators. For *p* = 0 and *p* = 1,we have

$$\begin{array}{c} \hat{f}\left(\eta ;0\right)=f_{0}\left(\eta \right),\;\hat{f}\left(\eta ;1\right)=f\left(\eta \right),\; \end{array}$$ (29)

$$\begin{array}{c} \hat{g}\left(\eta ;0\right)=g_{0}\left(\eta \right),\;\hat{g}\left(\eta ;1\right)=\hat{g}\left(\eta \right), \end{array}$$ (30)

$$\begin{array}{c} \hat{\theta }\left(\eta ,0\right)=\theta_{0}\left(\eta \right),\;\hat{\theta }\left(\eta ,1\right)=\theta \left(\eta \right), \end{array}$$ (31)

$$\begin{array}{c} \hat{\phi }(\eta ;0)=\phi_{0}(\eta ),\;\;\;\hat{\phi }(\eta ;1)=\phi (\eta ), \end{array}$$ (32)

and $\hat{f}(\eta ,p)$, $\hat{g}(\eta ,p),\hat{\theta }(\eta ,p)$ and $\hat{\phi }(\eta ,p)$ vary from *f*_0_(*η*), *g*_0_(*η*), *θ*_0_(*η*), *ϕ*_0_(*η*) to *f*(*η*), *g*(*η*), *θ*(*η*) and *ϕ*(*η*) when *p* varies from 0 to 1. By Taylor’s series expansion, we obtain

$$\begin{array}{c} \hat{f}\left(\eta ,p\right)=f_{0}\left(\eta \right)+\sum_{m=1}^{\infty }f_{m}\left(\eta \right)p^{m},f_{m}\left(\eta \right)=\frac{1}{m!}\frac{\partial^{m}\hat{f}\left(\eta ;p\right)}{\partial \eta^{m}}{|}_{p=0}, \end{array}$$ (33)

$$\begin{array}{c} \hat{g}\left(\eta ,p\right)=g_{0}\left(\eta \right)+\sum_{m=1}^{\infty }g_{m}\left(\eta \right)p^{m},\;g_{m}\left(\eta \right)=\frac{1}{m!}\frac{\partial^{m}\hat{g}\left(\eta ;p\right)}{\partial \eta^{m}}{|}_{p=0}, \end{array}$$ (34)

$$\begin{array}{c} \hat{\theta }\left(\eta ,p\right)=\theta_{0}\left(\eta \right)+\sum_{m=1}^{\infty }\theta_{m}\left(\eta \right)p^{m},\;\theta_{m}\left(\eta \right)=\frac{1}{m!}\frac{\partial^{m}\hat{\theta }\left(\eta ;p\right)}{\partial \eta^{m}}{|}_{p=0}, \end{array}$$ (35)

$$\begin{array}{c} \hat{\phi }(\eta ;p)=\phi_{0}(\eta )+\sum_{m=1}^{\infty }\phi_{m}(\eta )p^{m},\;\phi_{m}(\eta )=\frac{1}{m!}\frac{\partial^{m}\hat{\phi }(\eta ;p)}{\partial p^{m}}{|}_{p=0}, \end{array}$$ (36)

where the convergence of above series strongly depends upon *h*_*f*_, *h*_*g*_, *h*_*θ*_ and *h*_*ϕ*_. Considering that *h*_*f*_, *h*_*g*_, *h*_*θ*_ and *h*_*ϕ*_ are selected properly so that Eqs (34)–(36) converge at *p* = 1. Therefore

$$\begin{array}{c} \hat{f}\left(\eta \right)=f_{0}\left(\eta \right)+\sum_{m=1}^{\infty }f_{m}\left(\eta \right), \end{array}$$ (37)

$$\begin{array}{c} \hat{g}\left(\eta \right)=g_{0}\left(\eta \right)+\sum_{m=1}^{\infty }g_{m}\left(\eta \right), \end{array}$$ (38)

$$\begin{array}{c} \hat{\theta }\left(\eta \right)=\theta_{0}\left(\eta \right)+\sum_{m=1}^{\infty }\theta_{m}\left(\eta \right). \end{array}$$ (39)

$$\begin{array}{c} \hat{\phi }\left(\eta \right)=\phi_{0}\left(\eta \right)+\sum_{m=1}^{\infty }\phi_{m}\left(\eta \right). \end{array}$$ (40)

### 3.1 mth-order deformation problems

Differentiating the zeroth-order deformation problems m-times with respect to *p*, dividing by *m*! and then setting *p* = 0, we get *m*th order deformation problems in the following forms:

$$\begin{array}{c} {𝓛}_{f}[f_{m}\left(\eta \right)-\chi f_{m-1}\left(\eta \right)]=\hbar_{f}R_{f,m}\left(\eta \right), \end{array}$$ (41)

$$\begin{array}{c} {𝓛}_{g}[g_{m}\left(\eta \right)-\chi_{m}g_{m-1}\left(\eta \right)]=\hbar_{g}R_{g,m}\left(\eta \right), \end{array}$$ (42)

$$\begin{array}{c} {𝓛}_{\theta }[\theta_{m}\left(\eta \right)-\chi_{m\;}\theta_{m-1}\left(\eta \right)]=\hbar_{\theta }R_{\theta ,m}\left(\eta \right), \end{array}$$ (43)

$$\begin{array}{c} {𝓛}_{\phi }[\phi_{m}\left(\eta \right)-\chi_{m\;}\phi_{m-1}\left(\eta \right)]=\hbar_{\phi }R_{\phi ,m}\left(\eta \right), \end{array}$$ (44)

$$\begin{array}{ccc} f_{m}\left(0\right) & = & f_{m}^{\prime }\left(0\right)=f_{m}^{\prime }\left(\infty \right)=f_{m}^{\prime \prime }\left(\infty \right)\\ & = & g_{m}^{\prime }\left(0\right)=g_{m}^{\prime }\left(\infty \right)=g_{m}^{\prime \prime }\left(\infty \right)=0,\\ \theta_{m}^{\prime }\left(0\right)+\gamma \theta_{m}\left(0\right) & = & 0,\;\;\;\theta_{m}\left(\infty \right)=0,\phi_{m}(0)=\phi_{m}(\infty )=0. \end{array}$$ (45)

$$\begin{array}{c} R_{m}^{f}\left(\eta \right)=f_{m-1}^{\prime \prime \prime }-Kf_{m-1}^{\prime \prime \prime \prime \prime }-M^{2}f_{m-1}^{\prime }+\sum_{k=0}^{m-1}\left[\left(f_{m-1-k}+g_{m-1-k}\right)f_{k}^{\prime \prime }-f_{m-1-k}^{\prime }f_{k}^{\prime }\right], \end{array}$$ (46)

$$\begin{array}{c} R_{m}^{g}\left(\eta \right)=g_{m-1}^{\prime \prime \prime }-Kg_{m-1}^{\prime \prime \prime \prime \prime }-M^{2}g_{m-1}^{\prime }+\sum_{k=0}^{m-1}\left[\left(f_{m-1-k}+g_{m-1-k}\right)g_{k}^{\prime \prime }-g_{m-1-k}^{\prime }g_{k}^{\prime }\right], \end{array}$$ (47)

$$\begin{array}{ccc} R_{m}^{\theta }\left(\eta \right) & = & \theta_{m-1}^{\prime \prime }+\text{Pr}\sum_{k=0}^{m-1}[(f_{m-1-k}+g_{m-1-k})\theta_{k}^{\prime }]+2\mathrm{Pr}Ec\sum_{k=0}^{m-1}[(\begin{array}{c} f_{m-1-k}^{\prime \prime }f_{k}^{\prime \prime }+\\ L^{2}g_{m-1-k}^{\prime \prime }g_{k}^{\prime \prime } \end{array})]\\ & & -K\mathrm{Pr}Ec\sum_{k=0}^{m-1}[(\begin{array}{c} f_{m-1-k}^{\prime \prime \prime }f_{k}^{\prime \prime \prime }+\\ L^{2}g_{m-1-k}^{\prime \prime \prime }g_{k}^{\prime \prime \prime } \end{array})]+\mathrm{Pr}M^{2}Ec\sum_{k=0}^{m-1}[(\begin{array}{c} f_{m-1-k}^{\prime }f_{k}^{\prime }+\\ L^{2}g_{m-1-k}^{\prime }g_{k}^{\prime } \end{array})]\\ & & \mathrm{Pr}Nb\sum_{k=0}^{m-1}[\theta_{m-1-k}^{\prime }\phi_{k}^{\prime }]+\mathrm{Pr}Nt\sum_{k=0}^{m-1}[\theta_{m-1-k}^{\prime }\theta_{k}^{\prime }], \end{array}$$ (48)

$$\begin{array}{c} {𝓡}_{m}^{\phi }(\eta )=\phi_{m-1}^{\prime \prime }+Le\mathrm{Pr}\sum_{k=0}^{m-1}(f_{m-1-k}+g_{m-1-k})\phi_{k}^{\prime }+\frac{N_{t}}{N_{b}}\theta_{m-1}^{\prime \prime }, \end{array}$$ (49)

$$\begin{array}{c} \chi_{m}=\{\begin{array}{c} 0\;\;\;\;\;\;m\leq 1,\\ 1\;\;\;\;\;\;\;m>1. \end{array} \end{array}$$ (50)

### 3.2 Convergence analysis

**Fig 2:**
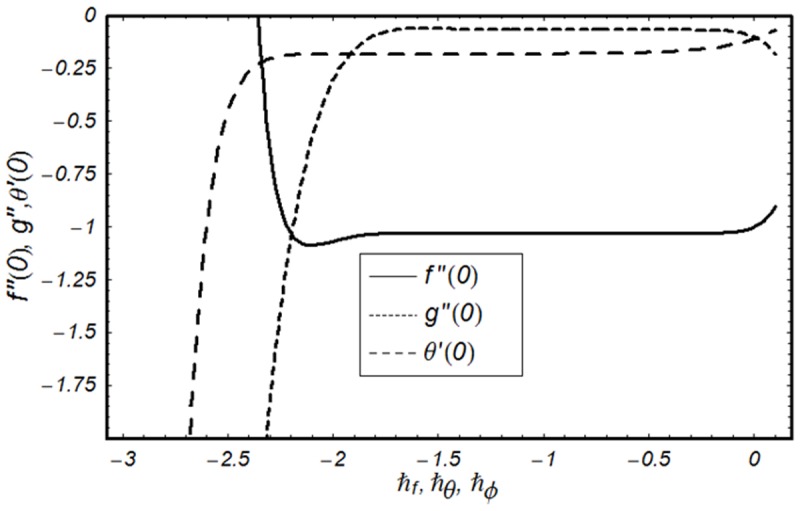
ℏ-curve for functions *f*, *g* and *θ*.

**Fig 3:**
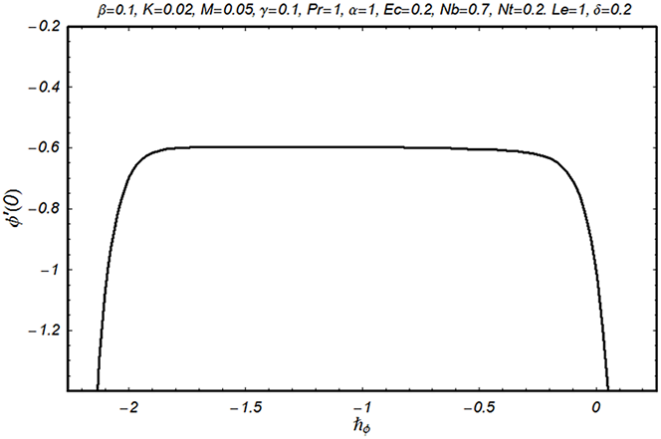
ℏ-curve for function *ϕ*.

**Fig 4:**
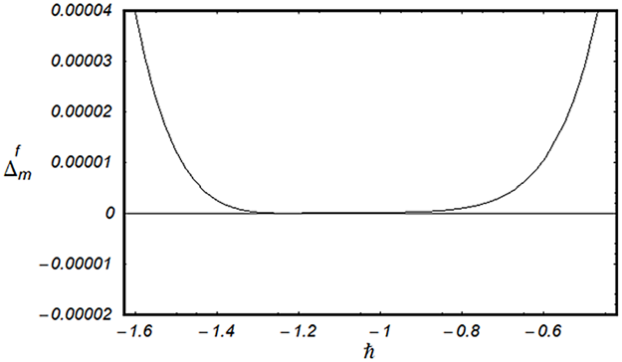
ℏ—curve for residual error $\Delta_{m}^{f}$.

**Fig 5:**
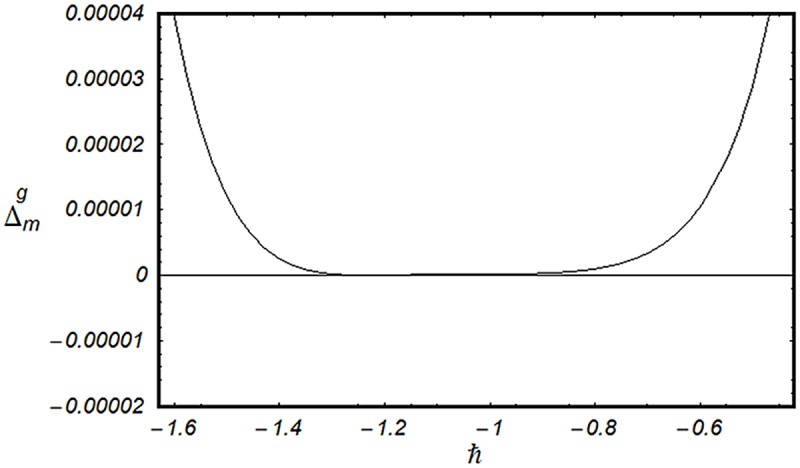
ℏ—curve for residual error $\Delta_{m}^{g}$.

**Fig 6:**
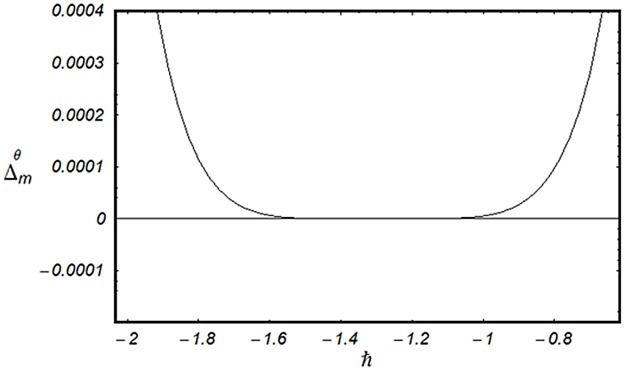
ℏ—curve for residual error $\Delta_{m}^{\theta }$.

**Fig 7:**
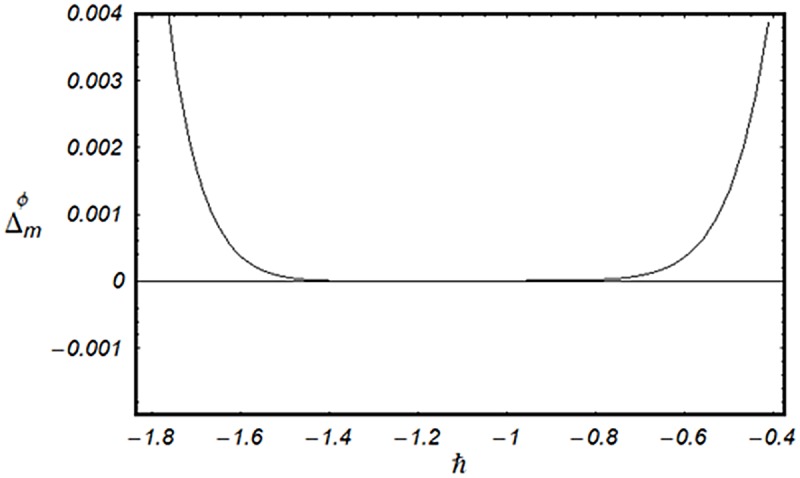
ℏ—curve for residual error $\Delta_{m}^{\phi }$.

This subsection aims to analyze the convergence of series solutions by homotopy analysis method (HAM). The HAM solutions contain the auxiliary parameter ℏ_*f*_, ℏ_*g*_, ℏ_*θ*_ and ℏ_*ϕ*_. Hence, the ℏ-curves are displayed for the convergence analysis. It is noticed that the admissible values of ℏ_*f*_, ℏ_*g*_, ℏ_*θ*_ and ℏ_*ϕ*_ are −1.4 ≤ ℏ_*f*_ ≤ −0.4, −0.9 ≤ ℏ_*g*_ ≤ −0.4, −2.0 ≤ ℏ_*θ*_ ≤ −0.6 and −1.6 ≤ ℏ_*ϕ*_ ≤ −0.6 (Figs 2 and 3). We have plotted the ℏ—curves for the square residual errors in Figs 4–7 when *β* = 0.1, *K* = 0.02, *M* = 0.05, *γ* = 0.1, Pr = 1.0, *α* = 1.0, *Ec* = 0.2, *Nb* = 0.7, *Nt* = 0.2, *Le* = 1.0 and *δ* = 0.2. The definition of square residual errors are [26, 27]:

$$\begin{array}{ccc} \Delta_{m}^{f} & = & \int_{0}^{1}{\left[{𝓡}_{m}^{f}(\eta ,\hbar_{f})\right]}^{2}d\eta ,\\ \Delta_{m}^{g} & = & \int_{0}^{1}{\left[{𝓡}_{m}^{g}(\eta ,\hbar_{g})\right]}^{2}d\eta ,\\ \Delta_{m}^{\theta } & = & \int_{0}^{1}{\left[{𝓡}_{m}^{\theta }(\eta ,\hbar_{\theta })\right]}^{2}d\eta ,\\ \Delta_{m}^{\phi } & = & \int_{0}^{1}{\left[{𝓡}_{m}^{\phi }(\eta ,\hbar_{\phi })\right]}^{2}d\eta , \end{array}$$ (51)

Fig 3 indicates that −1.25 ≤ ℏ_*f*_ ≤ −0.9 give us the lowest possible error. In the same manner, we have drawn the ℏ− curves for the residual errors of *g*, *θ* and *ϕ* in the Figs 5–7 respectively. It is noted that −1.25 ≤ ℏ_*g*_ ≤ −0.9, −1.5 ≤ ℏ_*θ*_ ≤ −1.05 and −1.35 ≤ ℏ_*ϕ*_ ≤ −0.9 certify the lowest possible errors. Figs 4–7 may be considered as counter check to Figs 2–3 in order to confirm their convergence analysis.

## 4 HAM-Based MATHEMATICA package BVPh 2.0

**Fig 8:**
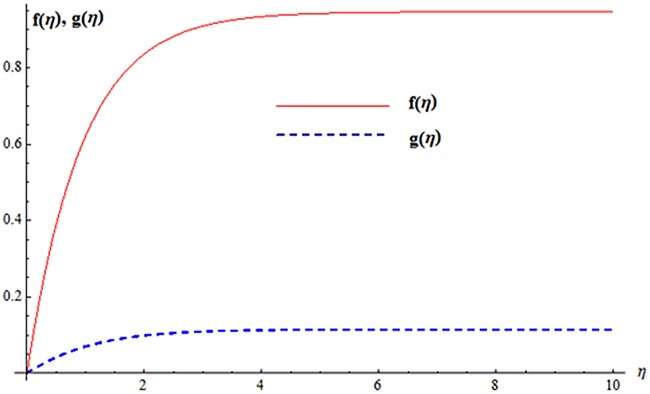
Solution curves for *f*(*η*) and *g*(*η*).

**Fig 9:**
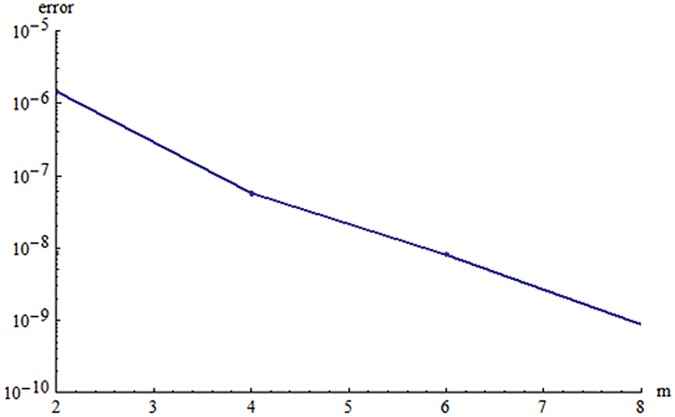
Total error vs. order of approximations.

We also computed the solution of nonlinear ordinary differential Eqs (8 and 9) by MATHEMATICA package BVPh 2.0 using the boundary condition (Eq 12). We have found the minimum squared residual errors for *f*(*η*) and *g*(*η*) are 5.602 × 10^−7^ and 9.70018 × 10^−6^ 8^th^ order of approximations. Fig 8 is plotted for the solution of velocity profiles. Total residual error corresponding to different order of approximations is displayed in Fig 9. It is noted that error decreases when order of approximation increases.

## 5 Discussion

**Fig 10:**
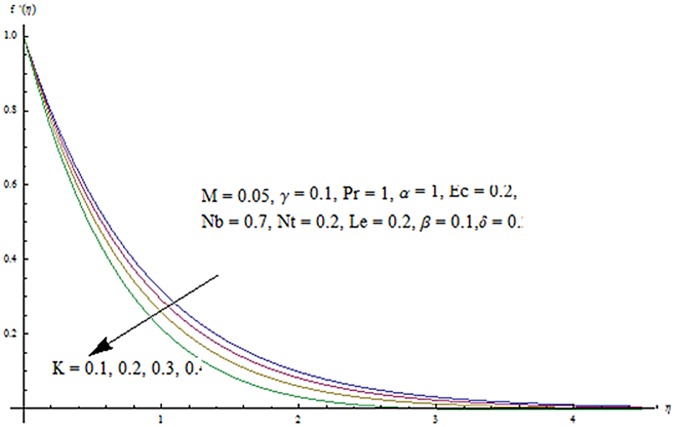
Influence of *K* on *f*′.

**Fig 11:**
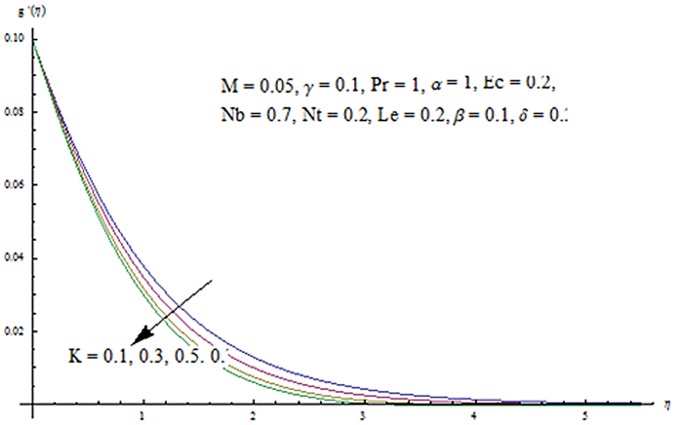
Influence of *K* on *g*′.

**Fig 12:**
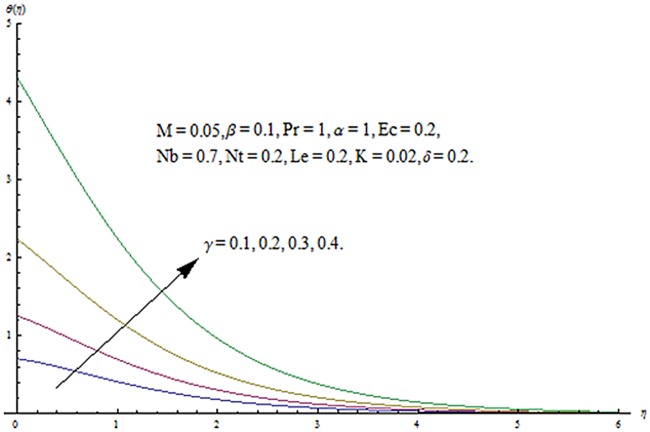
Influence of *γ* on *θ*.

**Fig 13:**
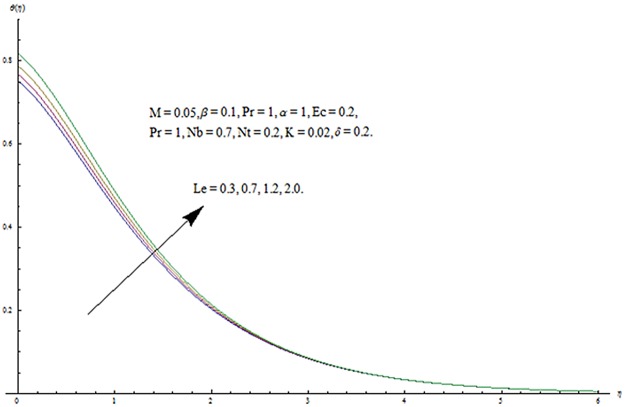
Influence of *Le* on *θ*.

**Fig 14:**
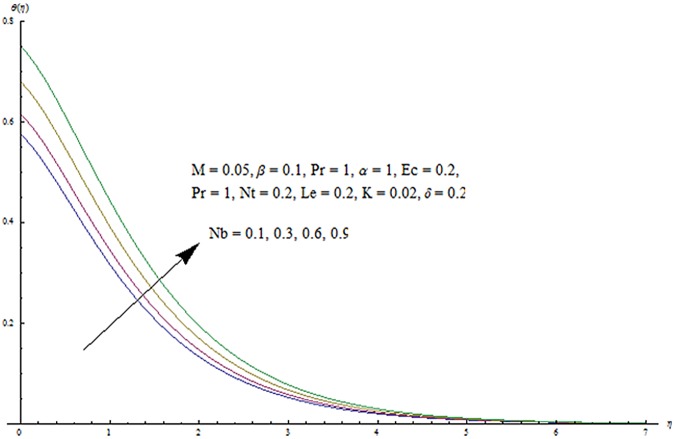
Influence of *Nb* on *θ*.

**Fig 15:**
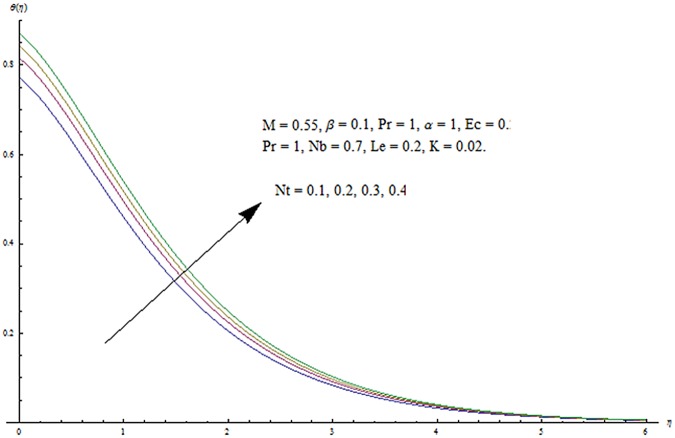
Influence of *Nt* on *θ*.

**Fig 16:**
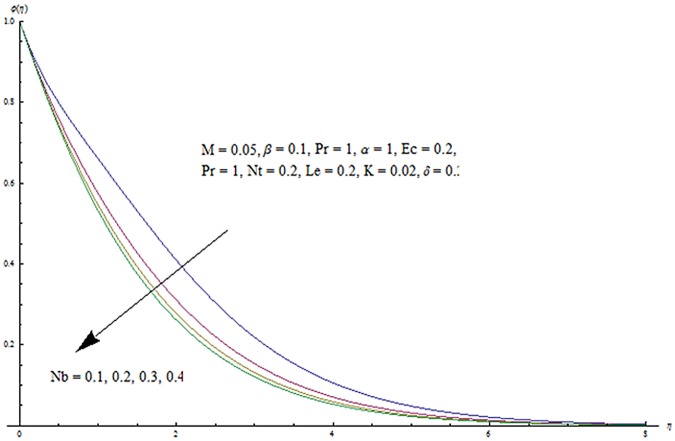
Influence of *Nb* on *ϕ*.

**Fig 17:**
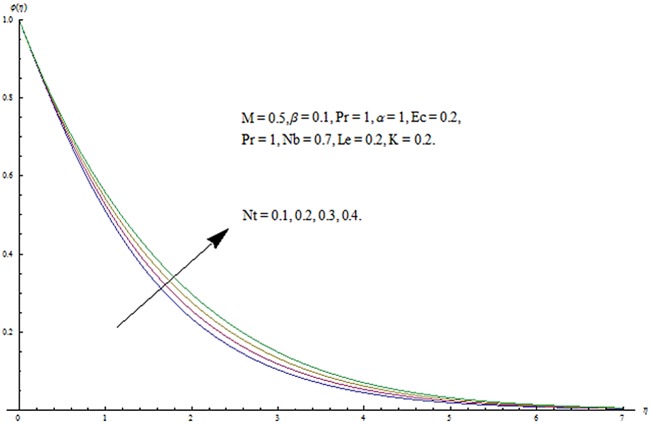
Influence of *Nt* on *ϕ*.

**Fig 18:**
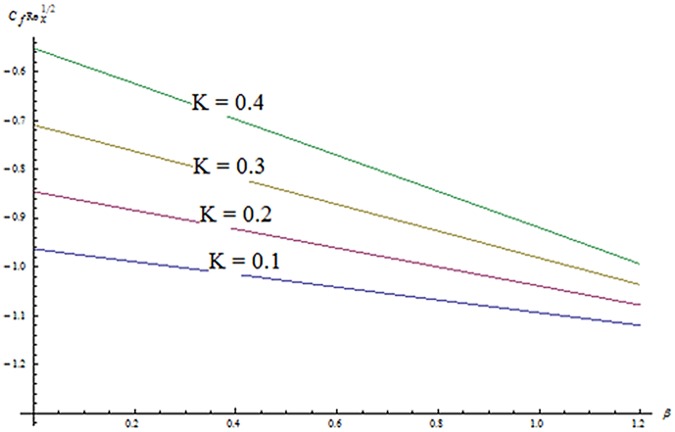
Effects of *K* and *β* on skin friction.

**Fig 19:**
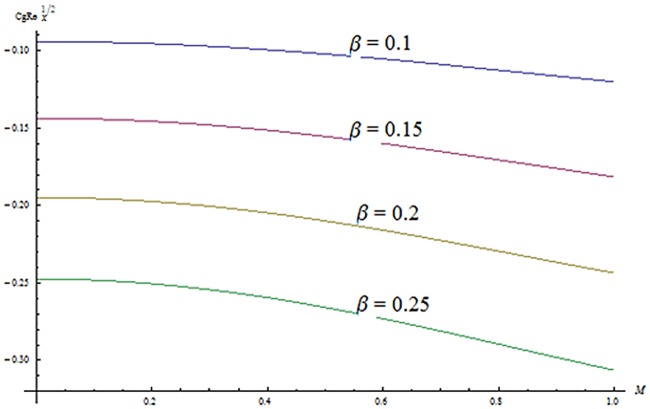
Effects of *β* and *M* on skin friction.

**Fig 20:**
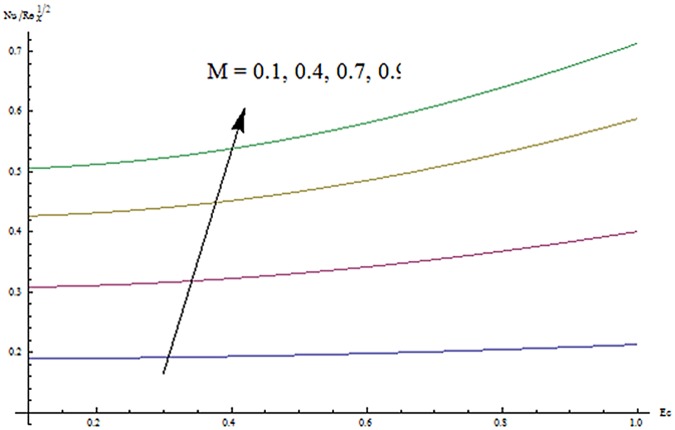
Effects of *M* and *Ec* on -*θ* ^′^(0).

**Fig 21:**
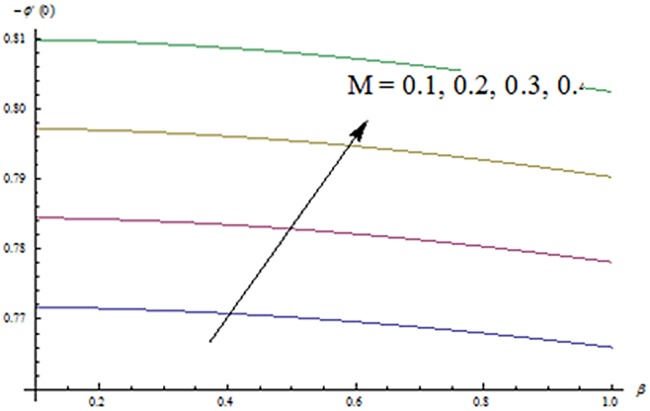
Effects of *M* and *β* on -*ϕ* ^′^(0).

The purpose of this section is to describe the salient features of emerging parameters on the velocity components and temperature. In all the Figures and Tables, we have used ℏ_*f*_ = ℏ_*g*_ = ℏ_*θ*_ = ℏ. The behavior of couple stress parameter *K* on velocity components is shown in Figs 10 and 11. It is observed that *f* ^′^ and *g* ^′^ decrease when couple stress parameter is increased. Couple stress parameter related to the viscosity *n* makes the fluid more viscous and consequently the velocity retarded. Influence of conjugate parameter for Newtonian heating *γ* on temperature profile is plotted in Fig 12. Here temperature profile *θ* increase with an increase in conjugate parameter. The thermal boundary layer is also an increasing function of conjugate parameter. Conjugate parameter increases the heat transfer coefficient which increases the temperature of the fluid. It is also noted that *γ* = 0 corresponds to insulated wall while *γ* → ∞ represents the constant wall temperature. Higher values of conjugate parameter results in the higher rate of heat transfer. So conjugate parameter can be used as a cooling agent in the advanced technological processes. Fig 13 reveals that as the Lewis number increases, the temperature and profile shows increasing behavior. Further thermal boundary layer thickness increases for larger Lewis number. It is the ratio of thermal diffusivity to mass diffusivity. In fact higher values of Lewis number results in more thermal diffusivity which is responsible in enhancement of temperature distribution. Fig 14 portraits the effects of Brownian motion parameter on temperature distribution. It is analyzed that temperature distribution is higher for larger values of Brownian motion parameter. As Brownian motion parameter *Nb* increases, random motion of the fluid particles increases which results in more heat to produce. Hence temperature profile increases. Characteristics of thermophoresis parameter *Nt* on temperature profile is sketched in Fig 15. Temperature profile and thermal boundary layer thickness are higher for larger values of thermophoresis parameter. It is a mechanism in which small particles are pulled away from hot surface to cold one. As a result it raises the temperature of the fluid. Behavior of Brownian motion parameter *Nb* on concentration distribution is displayed in Fig 16. It is interpreted that with the increase in *N*_*b*_ the random motion and also collision of the macroscopic particles of the fluid increases which reduces the concentration of the fluid. Effect of thermophoresis parameter *Nt* on concentration distribution is shown in Fig 17. It is depicted that as the value of *N*_*t*_ increases more nanoparticles are pushed away from the hot surface. So as a result the volume fraction distribution increases. Fig 18 shows the behavior of *β* and *K* on skin friction coefficient (*c*_*f*_ Re^1/2^). It is analyzed that skin friction coefficient increases for higher values of *β* while decreases with an increase in *K*. Characteristics of *β* and *M* on skin friction coefficients (*c*_*g*_ Re^1/2^) are displayed in Fig 19. Skin friction coefficient increases for higher values of *β* and *M*. Fig 20 represents the effect of *M* and *Ec* on Nusselt number. It is noted that Nusselt number is higher for larger values of *M* and *Ec*. Influence of *M* and *β* on Sherwood number is sketched in Fig 21. It is analyzed that Sherwood number increases for higher values of *M* while it decreases with *β*.

Table 1 represents the convergence of the series solutions for momentum, energy and concentration equations. It is noted that series solutions converge at 10th order of approximation for *f* ^′′^(0), 25th order of approximation for *g* ^′′^(0), 27th order of approximation for *θ* ^′^(0) and 30th order of approximation for *ϕ* ^′^(0). Tables 2 and 3 present the numerical values of skin friction coefficients. Table 4 shows the numerical values of Nusselt number for the different physical parameters. It is observed that local Nusselt number increases with the increase of *γ* and *δ*. However it decreases when values of *β*, *M*, Pr, *α*, *K*, *Ec*, *Nb*, *Nt*, *Le* and *K* are increased. Table 5 depicts the numerical values of local Sherwood number for different physical parameters keeping *α* and *δ* fixed. It is noticed that the values of Sherwood number increases with increase in values of with *β*, Pr, *Ec*, *Nb* and *Le* and decrease with increase in values of *K*, *M* and *Nt*. Tables 6–8 represent the comparison of skin friction coefficients and Nussselt number with the previous published data. It is analyzed that all the results are in good agreement.

**Table 1:** Convergence of series solution for different order of approximations when *β* = 0.1, *K* = 0.02, *M* = 0.05, *γ* = 0.1, Pr = 1, ℏ_*f*_ = ℏ_*g*_ = ℏ_*θ*_ = −0.5.

Order of approximations	−*f* ^′′^(0)	−*g* ^′′^(0)	−*θ* ^′^(0)	−*ϕ* ^′^(0)
1	1.0251	0.086396	0.13555	0.63421
5	1.0302	0.068033	0.17030	0.60835
10	1.0306	0.065947	0.17825	0.59918
15	1.0306	0.065896	0.17956	0.59735
20	1.0306	0.065899	0.17981	0.59702
25	1.0306	0.065903	0.17986	0.59694
27	1.0306	0.065903	0.11936	0.59695
30	1.0306	0.065903	0.17986	0.59695

**Table 2:** Numerical values of skin friction coefficient ($C_{f}Re_{x}^{1/2}$) for different parameters.

*β*	*K*	*M*	$C_{f}Re_{x}^{1/2}$
0.0	0.02	0.05	-0.99095
0.2			-1.0315
0.5			-1.0870
0.2	0.0		-1.0407
	0.02		-1.0315
	0.05		-1.0161
	0.02	0.0	-1.0304
		0.3	-1.0707
		0.5	-1.1388

**Table 3:** Numerical values of skin friction coefficient ($C_{g}Re_{x}^{1/2}$) for different parameters.

*β*	*K*	*M*	$C_{g}Re_{x}^{1/2}$
0.1	0.02	0.05	-0.067040
0.2			-0.14904
0.3			–0.24358
0.2	0.01		-0.14902
	0.02		-0.14904
	0.03		-0.14905
	0.2	0.01	-0.14873
		0.03	-0.14883
		0.05	-0.14914

**Table 4:** Values of local Nusselt number $\gamma (1+\frac{1}{\theta (0)})$ for different values of the parameters *β*, *M*, *γ*, Pr, *α*, *Ec*, *Nb*, *Nt*, *Le*, *δ* and *K*.

*β*	*M*	*γ*	*Pr*	*α*	*Ec*	*Nb*	*Nt*	*Le*	*δ*	*K*	$\gamma (1+\frac{1}{\theta (0)})$
0.1	0.05	0.1	1.0	1.0	0.2	0.7	0.2	1.0	0.2	0.02	0.2253
0.3											0.2337
0.5											0.2324
0.1	0.04										0.2254
	0.06										0.2253
	0.08										0.2250
	0.05	0.2									0.3054
		0.4									0.4004
		0.6									0.6000
		0.1	1.1								0.2231
			1.3								0.2172
			1.5								0.2095
			1.0	1.1							0.2253
				1.3							0.2250
				1.5							0.2246
0.1	0.05	0.1	1.0	1.0	0.1	0.7	0.2	1.0	0.2	0.02	0.2811
					0.3						0.1950
					0.5						0.1627
					0.1	0.4					0.3217
						0.6					0.2942
						0.8					0.2684
						0.7	0.1				0.2849
							0.3				0.2771
							0.5				0.2686
							0.2	1.1			0.2778
								1.3			0.2726
								1.5			0.2683
								1	0.1		0.2767
									0.3		0.2856
									0.5		0.2955
									0.2	0.00	0.2819
										0.03	0.2803
										0.05	0.2787

**Table 5:** Values of local Sherwood number −*ϕ* ^′^(0) for different values of the parameters *β*, *K*, *M*, *γ*, *α*, *Ec*, *Nb*, *Nt*, *Le*, *δ* and *K*.

*β*	*K*	*M*	*Pr*	*Ec*	*Nb*	*Nt*	*Le*	−*ϕ* ^′^(0)
0.2	0.02	0.05	1.0	0.1	0.7	0.2	1.0	0.6291
0.4								0.6890
0.6								0.7424
0.1	0.01							0.5964
	0.03							0.5943
	0.05							0.5923
	0.02	0.02						0.5956
		0.04						0.5955
		0.06						0.5952
			1.1					0.6378
			1.3					0.7179
			1.5					0.7954
0.2	0.02	0.05	1.0	0.1	0.7	0.2	1.0	0.5954
				0.2				0.5971
				0.4				0.6006
				0.6				0.6037
				0.1	0.4			0.5795
					0.6			0.5918
					0.8			0.5979
					0.7	0.1		0.6058
						0.3		0.5850
						0.5		0.5638
						0.2	1.1	0.6372
							1.3	0.7157
							1.5	0.7883

**Table 6:** Comparison of *f* ^′′^(0) and *g* ^′′^(0) with HPM and exact solutions [28] in limiting case for *K* = *M* = 0.

*β*	HPM [28]		Exact [28]		HAM	
	−*f* ^′′^(0)	−*g* ^′′^(0)	−*f* ^′′^(0)	−*g* ^′′^(0)	−*f* ^′′^(0)	−*g* ^′′^(0)
0.0	1.0	0.0	1	0	1.0	0.0
0.1	1.017027	0.073099	1.020260	0.066847	1.0203	0.066847
0.2	1.034587	0.158231	1.039495	0.148737	1.0395	0.14874
0.3	1.052470	0.254347	1.057955	0.243360	1.0580	0.24336
0.4	1.070529	0.360599	1.075788	0.349209	1.0758	0.34921
0.5	1.088662	0.476290	1.093095	0.465205	1.0931	0.46520
0.6	1.106797	0.600833	1.109947	0.590529	1.1099	0.59053
0.7	1.124882	0.733730	1.126398	0.724532	1.1264	0.72454
0.8	1.142879	0.874551	1.142489	0.866683	1.1425	0.86668
0.9	1.160762	1.022922	1.158254	1.016539	1.1582	1.01650
1.0	1.178511	1.178511	1.173721	1.173721	1.1737	1.17370

**Table 7:** Comparison of skin friction coefficient ($C_{f}Re_{x}^{1/2}$) for different parameters with Ramzan et al. [11].

*β*	*K*	*M*	Ramzan et al. [11]	Present results
0.0	0.2	0.1	-0.8515	-0.8515
0.2			-0.9268	-0.9268
0.5			-1.025	-1.025
0.2	0.0		-1.032	-1.032
	0.2		-0.9268	-0.9268
	0.5		-0.5664	-0.5664
	0.2	0.0	-0.8846	-0.8846
		0.3	-0.8938	-0.8938
		0.5	-0.8952	-0.8952

**Table 8:** Comparison of local Nusselt number $\gamma (1+\frac{1}{\theta (0)})$ for different values of the parameters *β*, *K*, *M*, *γ*, and *Pr* when *Nt* = *Nb* = 0.

*β*	*M*	*γ*	*Pr*	*α*	*Ec*	Ramzan et al. [11]	Present Results
0.1	0.05	0.1	1.0	1.0	0.2	0.28832	0.28832
0.3						0.29641	0.29641
0.5						0.29133	0.29133
0.1	0.04					0.28847	0.28847
	0.06					0.28814	0.28814
	0.08					0.28769	0.28769
	0.05	0.2				0.42247	0.42247
		0.4				0.55054	0.55054
		0.6				0.61873	0.61873
		0.1	1.1			0.29241	0.29241
			1.3			0.29823	0.29823
			1.5			0.30179	0.30179
			1.0	1		0.28813	0.28813
				1.3		0.28771	0.28771
				1.5		0.28722	0.28722
				1.0	0.1	0.37593	0.37593
					0.3	0.24294	0.24294
					0.5	0.19645	0.19645

## 6 Conclusions

This study develops the series solutions for MHD three dimensional flow of couple stress nanofluid. The main findings are summarized as follows:

- The impact of the Brownian motion *Nb* on the temperature and concentration fields are opposite.
- Effects of Lewis number *Le* and the Newtonian heating parameter *γ* on temperature profile are same.
- Velocity components *f* ^′^ and *g* ^′^ decrease with an increase in couple stress parameter *K*.
- The impact of the Brownian motion parameter *Nb* and the Lewis number *Le* on the temperature field is similar.
